# Transgenic Carrot Expressing Fusion Protein Comprising *M. tuberculosis* Antigens Induces Immune Response in Mice

**DOI:** 10.1155/2015/417565

**Published:** 2015-04-09

**Authors:** Natalia V. Permyakova, Alla A. Zagorskaya, Pavel A. Belavin, Elena A. Uvarova, Olesya V. Nosareva, Andrey E. Nesterov, Anna A. Novikovskaya, Evgeniy L. Zav'yalov, Mikhail P. Moshkin, Elena V. Deineko

**Affiliations:** ^1^Institute of Cytology and Genetics, Russian Academy of Sciences, Prospect Lavrentieva 10, Novosibirsk 630090, Russia; ^2^State Research Center of Virology and Biotechnology Vector, Koltsovo, Novosibirsk 630559, Russia

## Abstract

Tuberculosis remains one of the major infectious diseases, which continues to pose a major global health problem. Transgenic plants may serve as bioreactors to produce heterologous proteins including antibodies, antigens, and hormones. In the present study, a genetic construct has been designed that comprises the *Mycobacterium tuberculosis* genes *cfp10, esat6* and *dIFN* gene, which encode deltaferon, a recombinant analog of the human *γ*-interferon designed for expression in plant tissues. This construct was transferred to the carrot (*Daucus carota* L.) genome by *Agrobacterium*-mediated transformation. This study demonstrates that the fusion protein CFP10-ESAT6-dIFN is synthesized in the transgenic carrot storage roots. The protein is able to induce both humoral and cell-mediated immune responses in laboratory animals (mice) when administered either orally or by injection. It should be emphasized that *M. tuberculosis* antigens contained in the fusion protein have no cytotoxic effect on peripheral blood mononuclear cells.

## 1. Introduction

A highly efficient vaccine against* Mycobacterium tuberculosis* is in great demand because tuberculosis is currently among the diseases with the highest mortality rate. Every minute, approximately 20 people worldwide are infected with tuberculosis, and as a rule, four of them die [1]. Since the early 1990s, the mortality rate among tuberculosis patients has been steadily increasing, which is the result of increase in both drug-resistant* M. tuberculosis* strains and HIV infection [2]. Animals are also susceptible to tuberculosis. In particular, bovine tuberculosis, which is caused by the closely related bacterium* M. bovis*, is also hazardous to humans. Approximately 10% of tuberculosis patients were infected by animals [3].

Currently, the BCG vaccine, which has been in use for over 100 years, is the major tool for immunoprophylaxis of tuberculosis. Despite certain shortcomings of this vaccine [1], there is yet no alternative for immune prevention of this disease. This fact emphasizes the need for the development of new tools for tuberculosis prevention. Subunit vaccines that involve recombinant antigens in combination with cytokines are promising for this purpose [1, 4]. The most appropriate candidate subunit vaccines are antigens that activate CD4^+^ and CD8^+^ T cells and induce protective immunity. Currently, the* M. tuberculosis* secreted and cell wall proteins (ESAT6, Ag85B, MTB72F, and LipY) are regarded as the most promising antigens [5–7].

Secreted proteins [8] are of special interest among the antigens with a protective activity. ESAT6 (early secreted antigenic target, 6 kDa) and CFP10 (culture filtrate protein, 10 kDa) [5, 7] are the most valuable of these proteins. CFP10 is a chaperone for the ESAT6 protein, and together, they form heterodimeric complexes. CFP10 and ESAT6 are among the most important* M. tuberculosis* proteins involved in pathogen-host interactions [9, 10]. High immunogenicity and specificity of CFP10 and ESAT6 are confirmed by a high level of *γ*-interferon synthesis by peripheral blood mononuclear cells in response to contact with these antigens [11]. These proteins are encoded in the* M. tuberculosis* RD1 genomic region. Of note, this region is deleted in the* M. bovis* genome. Correspondingly, BCG administration fails to induce the immune response to the antigens encoded in this region, including CFP10 and ESAT6.

To increase the mucosal immunogenicity of proteins or peptides otherwise imperceptible to the mucosal immune system, recombinant antigens may be fused with proteins that display adjuvant or immunomodulatory properties [12]. A promising protein possessing such properties is deltaferon. This is a recombinant analog of *γ*-interferon with a deletion of ten C-terminal amino acids and mutations introduced at positions 129 and 130. As a result, deltaferon is synthesized in a native soluble form, which displays an increased resistance to blood proteases and a decreased antiviral activity [13, 14]. Therefore, deltaferon is able to concurrently serve as an adjuvant and an immunomodulator, efficiently boosting the immune response to tuberculosis antigens [15].

Various systems are used for delivering antigens as subunit vaccines to human and animal organisms, including recombinant viral vectors [16], recombinant bacterial systems [17], and lipoglycans conjugated with proteins [18]. Oral administration within plant cells [7, 19, 20] is among the promising systems. Proteins from plant cells expressing vaccine antigens are protected in the stomach from acids and enzymes but are released to the immune or blood circulatory system when plant cell walls are digested by microbes that colonize the gut. Vaccine antigens bioencapsulated in plant cells, provide both mucosal and systemic immunity and protection against bacterial, viral or protozoan pathogens or toxin challenge upon oral delivery. This new platform offers a low cost alternative to deliver different therapeutic proteins to combat infection by eliminating inactivated pathogens, expensive purification, cold storage/transportation, and sterile injections. We have used this particular approach when constructing transgenic carrot plants that produce* M. tuberculosis* antigens. Because the tuberculosis pathogen is a respiratory agent, mucosal vaccination is well suited to initiate both the mucosal and systemic immune responses.

We have previously produced transgenic carrot plants expressing CFP10 and ESAT6 individual proteins. It has been shown that oral immunization of mice with such CFP10 or ESAT6 induces both the cell-mediated and humoral immunities but it has also been demonstrated that they are toxic to peripheral blood mononuclear cells [21]. In the work of other researchers it was shown that vaccination with a fusion protein consisting of Ag85B and ESAT6 in animal and human models was more effective than either antigen alone [22]. Thus to increase the immune response and to decrease cytotoxic effect, we designed a new genetic construct comprising the fusion protein consisting of the* cfp10* and* esat6* genes of* M. tuberculosis* and the* dIFN* gene, encoding human deltaferon as adjuvant.* Agrobacterium*-mediated transformation was used to transfer this construct into the carrot (*Daucus carota *L.) genome. The fusion protein CFP10-ESAT6-dIFN was synthesized in the storage roots of transgenic carrot plants and displayed immunogenicity in the tests with laboratory animals. In this paper, we describe results for the studies focused on the synthesis of the fusion protein CFP10-ESAT6-dIFN in transgenic carrot plants followed by oral administration to animals.

## 2. Materials and Methods

### 2.1. Design and Assembly of Fusion Gene

The* esat6* and* cfp10* genes were cloned using genomic DNA extracted from biomass of an* M. tuberculosis* isolate recovered from a tuberculosis patient meeting the bioethical requirements. The protocol of that study was approved by the Committee on the Ethics, of State Research Center Virology and Biotechnology “Vector,” Koltsovo, Novosibirsk region, Russia, #IRB00001360, registered in OHRP, USA (Permit Number: #2, 28/05/2001). DNA was isolated with a DNeasy Blood and Tissue Kit (Qiagen, Germany) according to the manufacturer's protocol. The *γ*-interferon gene was amplified from the pIFN-*γ*-trp2-Δ plasmid [13].

The following primers (up1–lo3) were used for cloning the hybrid gene into various vectors:

 up1 CCCGGATCCATGGCAGAGATGAAGACCGAT;
 up2 GCTTCGGCGCGGGGATGACAGAGCAGCAGTGGAATT;
 up3 TCGCAGGCGCGGGGATGCAGGACCCATATGTAAAAGAA;
 up cfp-XbaI CCCTCTAGAATGGCAGAGATGAAGACCGAT;
 lo1 TGTCATCCCCGCGCCGAAGCCCATTTGCGAGGA;
 lo2 TGCATCCCCGCGCCTGCGAACATCCCAGTGACG;
 lo3 CCCAAGCTTACTGGGATGCTCTTCGACCT;
 lo esat-BamHI CCCGGATCCACTGGGATGCTCTTCGACCT.

**Figure 1:**
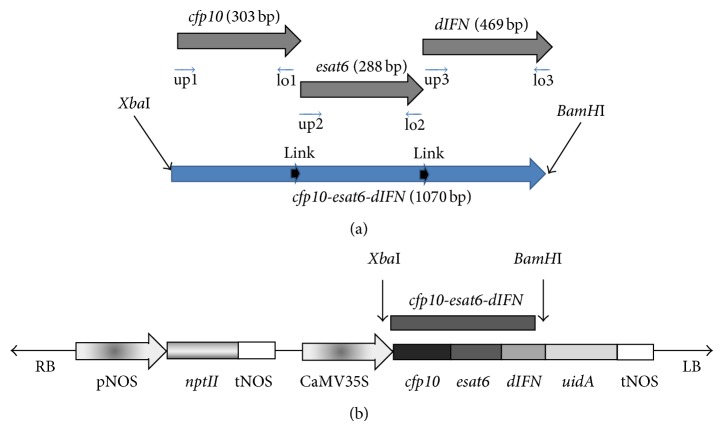
Schemes for assembly of the fusion gene (a) and for the T region in the binary plasmid pBI121 carrying sequence of the fusion gene* cfp10*-*esat6-dIFN *(b).* cfp10*, nucleotide sequence of the* M. tuberculosis cfp10 *gene;* esat6, *sequence of the* M. tuberculosis esat6 *gene;* dIFN,* sequence of the human deltaferon gene;* cfp10-esat6-dIFN,* sequence of the fusion gene;* nptII, E. coli* neomycin phosphotransferase II gene; and* uidA, *sequence of the* E. coli β*-glucuronidase gene. RB and LB are the repeats flanking the plasmid T region; pNOS and tNOS, nopaline synthase gene of the* A. tumefaciens* Ti plasmid promoter and terminator correspondingly; CaMV35S, cauliflower mosaic virus 35S RNA gene promoter. Link, gly–ala–gly hinge joints; and up1, up2, up3, lo1, lo2, and lo3 denote the positions of the corresponding primers. Arrows show the sites for *BamH*I and* Xba*I restriction endonucleases. The lengths of the corresponding gene sequences in base pairs are in parentheses.

Figure 1(a) shows the scheme for assembly of the hybrid gene* cfp10*-*esat6-dIFN.* The primers specific for* cfp10, esat6,* and* dIFN* genes were designed so that they would overlap and allow a single continuous “fusion” gene to be synthesized from the individual fragments. The primers that carry mutually complementary sequences at their 5′ ends allowed a glu–ala–glu link joint to be introduced. The presence of hybrid gene inserts was confirmed by PCR, and the absence of mutations in the produced clones was confirmed by sequencing at SB RAS Genomics Core Facility (data not shown).

In the plasmid pBI121-cfp10-esat6-dIFN, the fusion gene is under the control of the cauliflower mosaic virus 35S RNA promoter, which provides for its expression in plant cells. The T-DNA region of the plasmid pBI121, pBI121-cfp10-esat6-dIFN, is shown in Figure 1(b).

### 2.2. Construction and Analysis of Transgenic Plants (PCR and Western Blot)

The callus tissues induced from zygotic carrot (*D. carota *L., cultivar Nantskaya) embryos were transformed with the use of agrobacteria [23]. The transformants were selected using the selective media supplemented with the antibiotic kanamycin (100 mg/L). The kanamycin resistant plants were grown in a hydroponic greenhouse at 25/16°C and a photoperiod of 16/8 (day/night) until development of the storage roots. The genomic DNA of recipient carrot plants was isolated using an Axygen (United States) kit for DNA extraction according to the manufacturer's protocol.

Presence of the target gene in the genome of the obtained transgenic carrot plants was confirmed by PCR using up1 and lo2 primers. The expected fragment length was 570 bp.

For Western blot analysis, storage roots (6 g) of at least five individual plants were used. The fusion protein CFP10-ESAT6-dIFN was visualized, according to the standard protocols for Western blot assay [24] using a Pierce ECL Western Blotting Substrate (United States) for chemiluminescent reaction. The rabbit polyclonal serum for visualizing has been raised against the recombinant antigen rESAT6. Concentrations of proteins and TSP in the storage roots were assayed according to Bradford [25]. The optical density of solutions was measured in an Eppendorf BioPhotometer plus (Germany).

### 2.3. Production of Polyclonal Antibodies to rESAT6

Male rabbits (Gray Giant) at an age of 6 months were immunized according to the following scheme: day 1, 100 *μ*g of rESAT6 with 500 *μ*L of CFA intracutaneously; day 14, 100 *μ*g of rESAT6 with 500 *μ*L of FIA subcutaneously; and days 28 and 42, 100 *μ*g of rESAT6 subcutaneously. Animals were bled (5–10 mL) from the auricular vein on days 28, 42, and 56. The sera were obtained by natural clotting with subsequent purification from the blood cells by centrifugation at 3000 rpm for 10 min. The serum samples were frozen and stored at –20°C.

### 2.4. Production of the Recombinant Proteins rCFP10, rESAT6, and rCFP10-ESAT6-dIFN in* E. coli* and Their Purification

The recombinant proteins rESAT6 and rCFP10 (fused with GST polypeptide), which were used in experiments as a control, were earlier produced in* E. coli* strain BL21(DE3). The recombinant proteins were affinity purified on Glutathione Sepharose 4B (Pharmacia, Sweden) [26]. The fusion recombinant protein rCFP10-ESAT6-dIFN (fused with His tag) was produced in* E. coli* strain BL21(DE3). Fusion gene expression was induced with 1 mM IPTG. The fusion protein was isolated and purified using a Ni-NTA Spin Kit (Qiagen, Germany) according to the manufacturer's protocol. The recombinant deltaferon was kindly provided by L. R. Lebedev (SRC VB Vector, Koltsovo, Novosibirsk oblast, Russia).

### 2.5. Laboratory Animals

The study was conducted at the Center for Genetic Resources of Laboratory Animals at the Institute of Cytology and Genetics, Siberian Branch, Russian Academy of Sciences (RFMEFI61914X0005 and RFMEFI61914X0010). This study was carried out in strict accordance with the recommendations in the Guide for the Care and Use of Laboratory Animals of the Russian National Center of Genetic Resources of Laboratory Animals based on SPF Vivarium of Institute of Cytology and Genetics SB RAS. The protocol was approved by the Committee on the Ethics of Animal Experiments of the Russian National Center of Genetic Resources of Laboratory Animals based on SPF Vivarium of Institute of Cytology and Genetics SB RAS, Novosibirsk, Russia (Permit Number: 10, July 3, 2012). All surgery was performed under halothane anesthesia and all efforts were made to minimize suffering.

SPF inbred BALB/c male mice at an age of 6-7 weeks used in the work were obtained from the SPF Vivarium with the Institute of Cytology and Genetics, Siberian Branch, Russian Academy of Sciences (Novosibirsk, Russia). The animals were kept in groups of two to three males per cage (Optimice, Animal Care Systems, Inc.). The cages were placed in a room with a 20-fold air exchange at a temperature of 24 ± 2°C, humidity of 45–50%, and artificial 12/12 (day/night) photoperiod with a daybreak at 0.3:00 a.m. The feed and litter were autoclaved at 121°C before use. Animals were provided with deionized water (produced in a Millipore device) with a Severyanka (Eko-proekt, St. Petersburg, Russia) mineral supplement and feed* ad libitum*.

#### 2.5.1. Experimental Model 1: Oral Antigen Administration

The mice were divided into four groups: three experimental groups of six individuals each and control group of three animals. The mice from the experimental groups were fed transgenic carrot storage roots containing the antigens CFP10, ESAT6, or rCFP10-ESAT6-dIFN on days 1 and 14 of experiment after a 12-h complete food deprivation. The control mice were fed nontransgenic storage roots. After 12 h, the unconsumed storage root remains were weighed. In the remaining days, the animals received standard feed* ad libitum*. Before feeding, the storage roots were processed as follows: all storage roots were exposed to *γ*-radiation (IGUR-1, Russia) with an intensity of 8400 R/h for 48 h (total irradiation dose, 400 kR), 5–7 storage roots were minced, and 10 g/male was placed into feedboxes.

#### 2.5.2. Experimental Model 2: Antigen Administration by Injection

Similar to experimental model 1, the mice were divided into four groups, three experimental (six individuals each) and one control (three individuals) groups. All animals were subcutaneously injected twice in the interscapular region with 50 *μ*L of antigen (rCFP10, rESAT6, or rCFP10-ESAT6-dIFN) with subsequent reimmunization after 14 days in the experimental groups or with 50 *μ*L of PBS in the control group.

### 2.6. Sampling and Sample Preparation

Animals were intravitally bled (200 *μ*L) from the retroorbital sinus on days 13 and 21 after the first immunization. On day 28, animals were euthanized by decapitation and blood was sampled. The blood specimens were divided into two portions: one portion was placed into tubes with anticoagulant (5% sodium EDTA solution) and the other portion into clean tubes without anticoagulant. The blood specimens with anticoagulant were used to isolate peripheral blood leukocytes for LTT (lymphocyte transformation test). The blood without anticoagulant was used to obtain blood serum through natural clotting. The serum specimens were frozen in Eppendorf tubes and stored at –80°C.

The humoral immune response was assayed by solid-phase immune assay of the antibody content in blood serum and the cell-mediated immune response was assayed by LTT.

The spleen was aseptically excised from euthanized mice and placed into RPMI 1640 nutrient medium (2 mM L-glutamine, 80 mg/L gentamicin, and 5% fetal bovine serum). The resulting splenocyte suspension was used to assess the proliferative activity of deltaferon and recombinant antigens.

### 2.7. Enzyme Immunoanalysis (EIA) Estimation of Humoral Immunity

Antibodies were detected in the blood specimens by solid-phase enzyme immunoassay [27]. To assess the humoral immunity, Corning plates were sensitized with solutions of the recombinant antigens (rESAT6, rCFP10, or rCFP10-ESAT6-dIFN). Nonspecific binding sites were saturated with 3% fetal bovine serum solution [27]. Wells of the plates prepared with recombinant antigens were supplemented with 50 *μ*L of the assayed blood samples. The reaction was stopped by adding 100 *μ*L/well of 0.9 M sulfuric acid and optical density was measured in an EnSpire multimode plate reader (United States) at a wavelength of 450 nm.

### 2.8. Estimation of Cell-Mediated Immunity by LTT

To isolate the peripheral blood mononuclear cells, the blood with anticoagulant was layered onto a Histopaque-1119 (Sigma, United States) gradient and centrifuged for 15 min at 500 rpm. The suspension of mononuclear cells was collected in a clean tube, washed with RPMI 1640, and suspended in the same medium.

Proliferative activity was assessed using the dye MTT (20 *μ*L), which was added 3 h before the end of cultivation according to the recommendations for TASC MTT Assays (R&D Systems, United States) [28]. We used the nonspecific proliferation inducers phytohemagglutinin (PHA; Sigma, United States) or concanavalin A (ConA, Sigma, United States) (positive control) as well as specific inducers, namely, the recombinant proteins dIFN, rESAT6, rCFP10, or rCFP10-ESAT6-dIFN. The cell proliferative activity was estimated according to the stimulation index (SI; the ratio of mean absolute OD values for stimulated cells to the mean absolute OD values for unstimulated cells).

### 2.9. Statistical Processing

Because blood was sampled for estimation of the immunogenic effects of the tested vaccines at three subsequent time points from each animal, the repeated-measures ANOVA test was used for normally distributed or log-transformed traits. The antigen administration route (feeding or injection) and the protein used as an antigen were the factors. Additionally, the experimental groups were compared with the controls using repeated-measures ANOVA. In this process, gradation of the antigen type factor was reduced to two levels, control and antigen. When analyzing the LTT data, Student's *t*-test was used to assess whether the SI exceeded unity as (SI − 1)/SE. The SE value was taken from the table of unweighted averages and their errors (SE) obtained by repeated-measures ANOVA. In the experiment with splenocytes, the SI difference from unity was calculated using the nonparametric sign test.

## 3. Results

### 3.1. Genetic Construct with Fusion Gene

The design of the construct makes it possible to synthesize the full-sized gene* cfp10-esat6-dIFN.* It is composed of two individual* M. tuberculosis* genes,* esat6* and* cfp10,* and the gene encoding human deltaferon (*dIFN*) within one open reading frame. There are no internal stop codons between them, but there is a “hinge joint” of three amino acid residues Gly–Ala–Gly that creates a flexible link between the domains of the fusion protein.

### 3.2. Analysis of Transgenic Carrot Plants

In the experiments, about 100 transgenic carrot plants carrying the gene* cfp10-esat6-dIFN* were obtained; the transgenic carrot plants with the* cfp10* and* esat6 *genes were obtained earlier [21].

**Figure 2:**
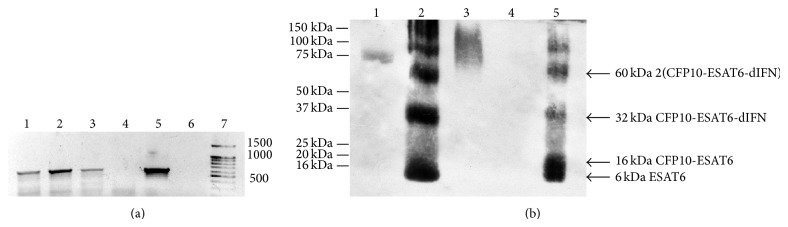
Analysis of transgenic carrot plants. (a) PCR reaction. Electrophoretic patterns of the PCR amplified genomic DNA of three selected transformants assayed for the presence of the fusion gene* cfp10-esat6-dIFN *(1.5% agarose gel): (1–3) DNA of transgenic carrot plants; (4) DNA of a nontransgenic carrot plant; (5) positive control (plasmid pBi121-CFP10-ESAT6-dIFN); (6) negative control (no template DNA); and (7) DNA length marker (bp). (b) Western blot assay of the extracts (total soluble protein) of transgenic carrot storage roots: In (b), lane (1) molecular weight marker Precision Plus Protein Kaleidoscope Standards (BioRad, USA); lane (2) control 1, purified rCFP10-ESAT6-dIFN protein produced in* E. coli*; lane (3) control 2, rCFP10-ESAT6-dIFN protein from inclusion bodies of* E. coli*; lane (4) negative control, extract of nontransgenic carrot storage roots; lane (5) extract of transgenic carrot storage roots. Molecular weights of the visualized fragments and their assumed composition are shown to the right; marker proteins molecular weights are shown to the left.

To confirm the transgenic status of the resulting carrot plants, the storage root of each one was tested for presence of the* cfp10-esat6-dIFN* nucleotide sequence in the genomic DNA by PCR with the corresponding primers. The electrophoretic patterns of PCR products for three carrot storage roots are shown in Figure 2(a). The length of the amplified fragments is consistent with the expected PCR fragment obtained from the plasmid (570 Bp) in lanes 1–3, as compared with the amplified positive control fragment in lane 5. This suggests that the hybrid gene* cfp10-esat6-dIFN *was integrated into the nuclear genome of the assayed carrot plants.

The Western blot assay was used to confirm the presence of hybrid protein in the storage roots of transgenic carrot plants. The polyclonal serum for visualizing the studied protein has been raised against the recombinant antigen rESAT6. The corresponding results are shown in Figure 2(b).

In Figure 2(b), lane (1) is loaded with molecular weight marker Precision Plus Protein Kaleidoscope Standards (BioRad, USA), with fluorescent band of 75 kDa. Lane (2) is loaded with purified rCFP10-ESAT6-dIFN protein (10 ng) produced in* E. coli, *as control 1. Lane (3) is loaded with rCFP10-ESAT6-dIFN protein from inclusion bodies of* E. coli *as control 2. Lane (4) is loaded with extract of nontransgenic carrot storage roots (22.320 *μ*g of TSP) as negative control. Lane (5) is loaded with extract of transgenic carrot storage roots carrying the gene* cfp10-esat6-dIFN *(28.140 *μ*g of TSP). Several bands visualized in lanes (2) and (5) of Figure 2(b) presumably correspond to the following peptides according to their molecular weights: ESAT6 (6 kDa), CFP10-ESAT6 (16 kDa), CFP10-ESAT6-dIFN (32 kDa), and a putative dimer or trimer of one of these species 2(CFP10-ESAT6-dIFN) (60 kDa).

The concentration of the fusion protein in carrot storage root tissues was compared with the known concentration of the protein isolated from* E. coli*. 20 *μ*L of TSP solution (TSP concentration of 1.407 *μ*g/*μ*L) of transgenic carrot storage roots was applied on membrane, so we applied 1.407∗20 = 28.140 *μ*g of TSP on membrane. Control sample was applied in amount of 10 ng. Therefore, in comparison with rCFP10-ESAT6-dIFN on lane (2) we see not less than 10 ng of ESAT6 on lane (5), suggesting that the CFP10-ESAT6-dIFN concentration in the extracts of transgenic carrot storage roots amounted to not less than 1 ng/*μ*L, which is not less than 0.035% TSP.

### 3.3. Establishment of Humoral Immunity

**Figure 3:**
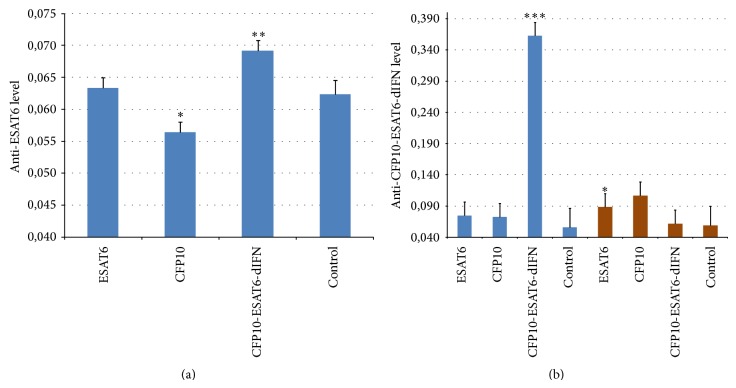
(a) Level of the antibodies to rESAT6 antigen in the blood serum of the mice immunized with different antigens: ^∗^ *F*_1.14_ = 5.14 and *P* = 0.039 and ^∗∗^ *F*_1.14_ = 12.05 and *P* = 0.0037; (b) level of the antibodies to rCFP10-ESAT6-dIFN with different methods of antigen presentation, blue bars, antigen delivered by injection, brown bars, antigen delivered orally: ^∗^ *F*_1.7_ = 6.55 and *P* = 0.038 and ^∗∗∗^ *F*_1.74_ = 276.10 and *P* < 0.001 both when compared with the control for the mouse groups immunized with the corresponding antigen (repeated-measures ANOVA, two gradations of control as a factor and one of the antigens).

Figure 3(a) shows the level of the antibodies to the rESAT6 antigen in blood sera of mice immunized with three types of antigens (rESAT6, rCFP10, and rCFP10-ESAT6-dIFN) that were delivered either by injection or orally. According to the results from a repeated-measures ANOVA, the efficiency of immunization with rESAT6 was independent of the administration route (*F*_1.34_ = 0.03, *P* = 0.86 for log-transformed data), which suggested pooling the data for oral and injection immunizations. However, the type of antigen was shown to be a significant factor for variation in the content of anti-rESAT6 antibodies in the blood of experimental animals (*F*_3.34_ = 12.39, *P* < 0.001 for log-transformed data). Compared with controls, immunization with rCFP10-ESAT6-dIFN elevated the level of antibodies to rESAT6 in mouse blood serum, whereas immunization with rCFP10 decreased this level (Figure 3(a)).

Independent of the administration route (oral or by injection), none of the tested antigens had a statistically significant effect on the content of antibodies to rCFP10 (data not shown).

A statistically significant effect of the antigen type (*F*_3.34_ = 11.82 and *P* < 0.001) and administration route (*F*_1.34_ = 7.61 and *P* = 0.009) as well as the interaction of these factors (*F*_3.34_ = 18.88 and *P* < 0.001) was demonstrated when analyzing the level of antibodies to rCFP10-ESAT6-dIFN (Figure 3(b)). Taking into account the significant dependence of the antibody level in the animal blood on the administration route, the data for antibodies to rCFP10-ESAT6-dIFN were analyzed separately for the animals immunized orally (brown bars) or by injection (blue bars).

The rCFP10-ESAT6-dIFN injections induced the maximal increase in the antibodies specific for this antigen in the mouse blood serum (Figure 3(b)). It should be emphasized that level of the antibodies to rCFP10-ESAT6-dIFN strikingly exceeded the control level (*F*_1.7_ = 276.10 and *P* < 0.001). The levels of antibodies to rCFP10 and rESAT6 in mice immunized with rCFP10-ESAT6-dIFN were at the control level (Figure 3(b)).

In the variant with oral rCFP10-ESAT6-dIFN administration, a statistically significant increase in the level of antibodies as compared with control was observed only when feeding animals with the carrot storage roots containing the rESAT6 protein (*F*_1.7_ = 6.55, *P* = 0.038). Of note, the induction of specific antibodies in the animal blood was undetectable in the case of oral rCFP10-ESAT6-dIFN administration (Figure 3(b)).

### 3.4. Cell-Mediated Immunity

Cell-mediated immunity was estimated using the stimulation index (SI) for proliferation of peripheral blood mononuclear cells induced by recombinant proteins (rESAT6, rCFP10, or rCFP10-ESAT6-dIFN) and nonspecific proliferation inducers (PHA or ConA).

**Figure 4:**
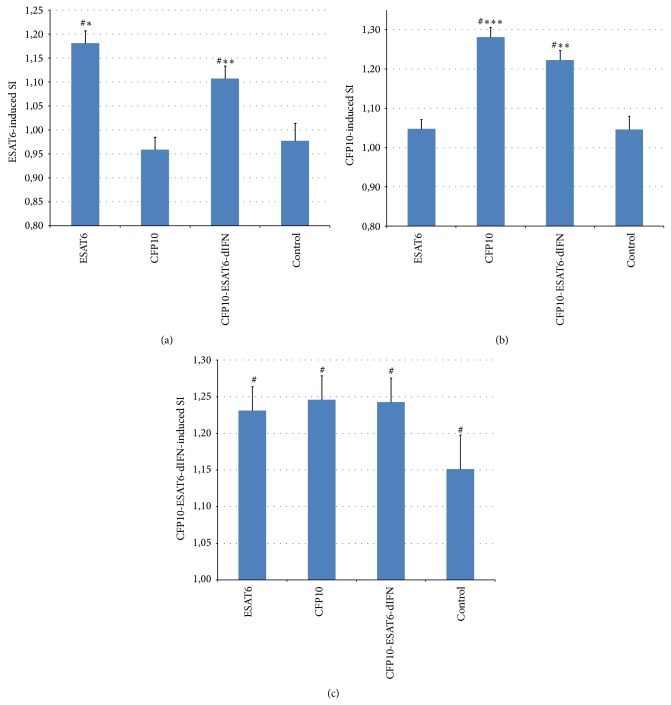
The SI for the proliferation of peripheral blood mononuclear cells induced by recombinant proteins after immunization with different antigens. (a) The SI for rESAT6: ^∗∗∗^ *F*_1.14_ = 15.36 and *P* = 0.0015 and ^∗∗^ *F*_1.14_ = 12.21 and *P* = 0.0036; (b) the SI for rCFP10: ^∗∗∗^ *F*_1.14_ = 32.58 and *P* = 0.001 and ^∗∗^ *F*_1.14_ = 11.63 and *P* = 0.0046; (c) the SI for rCFP10-ESAT6-dIFN. All *F*_1.14_ as compared to the control for the mouse group immunized with this antigen (repeated-measures ANOVA; administration route as a factor, two gradations of control as a factor, and one of the antigens); ^#^ *P* < 0.05 as compared with unity (Student's *t*-test).

The proliferative response of mononuclear cells to rESAT6 (Figure 4(a)) and to rCFP10 (Figure 4(b)) depended on the antigen used for immunization of experimental animals (*F*_3.34_ = 15.12 and *P* < 0.001 for rESAT6 and *F*_3.34_ = 20.97 and *P* < 0.001 for rCFP10). Proliferation was independent of the two administration routes, either by injection or orally (*F*_1.34_ = 0.00 and *P* = 0.99 for rESAT6 and *F*_1.34_ = 1.99 and *P* = 0.17 for rCFP10). Of note, the animals immunized with rESAT6, rCFP10, or rCFP10-ESAT6-dIFN either by injection or orally displayed a statistically significant increase in response as compared with the control.

The SI induced by the fusion protein (Figure 4(c)) added to the culture medium was independent of both administration route (*F*_1.34_ = 0.69 and *P* = 0.41) and type of antigen (*F*_3.34_ = 1.07 and *P* = 0.37). Notably, the SI for mononuclear cells of both control and immunized animals exceeded unity in a statistically significant manner, which could result from the presence of deltaferon in the fusion protein.

**Figure 5:**
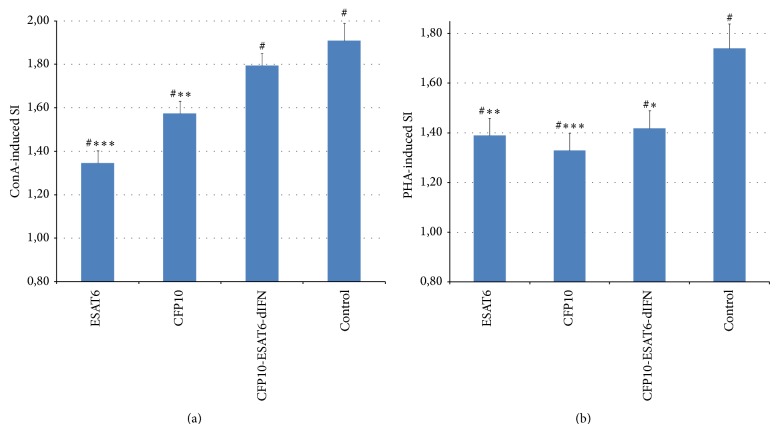
The SI for the proliferation of peripheral blood mononuclear cells induced by nonspecific proliferation inducers after immunization with different antigens: (a) the SI for ConA: ^∗∗∗^ *F*_1.14_ = 40.74 and *P* = 0.001 and ^∗∗^ *F*_1.14_ = 15.85 and *P* = 0.0013; (b) the SI for PHA: ^∗∗∗^ *F*_1.14_ = 13.87 and *P* = 0.002, ^∗∗^ *F*_1.14_ = 8.97 and *P* = 0.001, and ^∗^ *F*_1.14_ = 6.21 and *P* = 0.026. Both *F*_1.14_ as compared to the control for the mouse group immunized with this antigen (repeated-measure ANOVA; administration route as a factor, two gradations of control as a factor, and one of the antigens); ^#^ *P* < 0.05 as compared with unity (Student's *t*-test).

Addition of ConA (Figure 5(a)) or PHA (Figure 5(b)) to the culture medium induced a statistically significant proliferative response in all animals and was most pronounced in the control individuals. However, the degree of this response depended on the type of antigen used for immunization (*F*_3.34_ = 16.11 and *P* < 0.001 for ConA and *F*_3.34_ = 3.58 and *P* = 0.024 for PHA) rather than on the administration route (*F*_1.34_ = 1.23 and *P* = 0.27 for ConA and *F*_1.34_ = 1.74 and *P* = 0.20 for PHA). Immunization with rESAT6 or rCFP10 induced a statistically significant decrease in the SI as compared with control, whereas statistically significant differences were not observed in the case of the fusion protein.

### 3.5. Proliferative Response of Splenocytes

**Figure 6:**
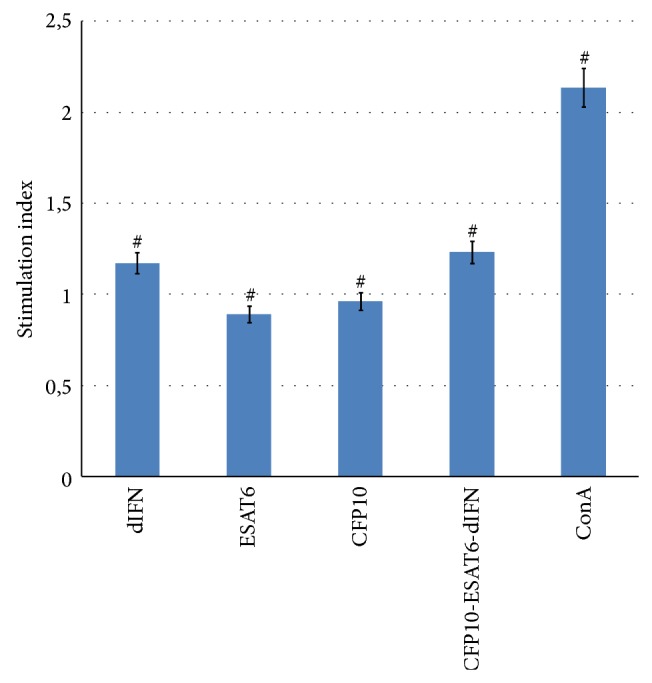
The SI for spontaneous proliferation of mouse splenocytes induced by different antigens: ^#^SI exceeds unity (*P* < 0.05, sign test).

Deltaferon, a component of the fusion protein rCFP10-ESAT6-dIFN, could be the factor that stimulated proliferation of mononuclear cells in the control animals that received no antigens (Figure 6). To test this assumption, we estimated the proliferative response of splenocytes isolated from intact male BALB/c mice (Figure 6). When dIFN and the fusion protein were added to the culture, the recorded SI exceeded unity in a statistically significant manner (*Z* = 2.04 and *P* = 0.04, sign test). The average SI values were equal for dIFN and rCFP10-ESAT6-dIFN but exceeded the values for splenocyte cultures that received rESAT6 or rCFP10.

## 4. Discussion

Currently, plant cells are an attractive alternative expression system for recombinant proteins with medical purposes and are used in many leading biotechnological laboratories and companies [29–31]. Recent advances in this field have enabled a significant increase in the expression level of recombinant proteins [32–34], improvement in posttranslational modifications that fit more closely with mammalian cells [35, 36], and approaches to directed modification of the plant genome [37, 38]. It has become evident that plant systems possess a high potential competitive ability compared with other expression systems and are of interest for investment companies.

There are examples of successful expression of* M. tuberculosis* antigens in plant cells [3, 7, 19, 20].

Cell-mediated and humoral immune responses induced by edible vaccines are formed via presentation of an antigen to the intestinal mucosae. Importantly, in most cases, lung mucosa is the particular site where tuberculosis infections starts and mainly progresses. Correspondingly, the immune response in lung tissues is a major factor in the initial stages of the disease development and colonization by* M. tuberculosis* and* M. bovis* in warm-blooded organisms [39]. The fact that the body mucosae function as an integral system, in which the activated lymphocytes and the corresponding interleukins circulate, allows us to speak to a unified mechanism that underlies establishment of mucosal immunity during disease development or administration of an edible vaccine.

The ESAT6 and CFP10 proteins, secreted at an early stage of tuberculosis, stimulate T cells to produce *γ*-interferon and exhibit CTL activity (cytotoxic T lymphocytes) both in animal models and in humans. According to recent data, these two proteins together have a high potential as a candidate subunit vaccine [5]. As has been experimentally shown, the efficiency of recombinant ESAT6 in inducing protective immunity against tuberculosis is comparable to the efficiency of the BCG vaccine [6].

A promising direction in the development of subunit vaccines is combining the antigens with adjuvants and immunomodulators. Plant-based vaccines targeting different diseases through the use of chimeric proteins as immunogens have been of great interest to vaccine developers. Progress over the past decade in the design and evaluation of new broad-protective proteins has demonstrated the feasibility of this technology. Genetic fusions allowed the expression of fusion proteins carrying two or more components with the aim to elicit immune responses against different targets, including antigens from distinct pathogens or strains. To increase immunogenicity, the ESAT6 antigen was fused with other tuberculosis antigens (Ag85B and Mtb72F) [3, 20, 40, 41] or adjuvants (CTB: cholera toxin B subunit, LTB:* Escherichia coli* heat-labile enterotoxin B subunit, LipY: a cell wall protein, and ELP: elastin-like peptide) [3, 7, 42] and expressed in various plant species (*Arabidopsis thaliana*, tobacco, and lettuce).

Earlier in another experiment we have assessed immunogenicity of the recombinant* M. tuberculosis* proteins ESAT6 and CFP10 in experiments with laboratory animals [21]. In this novel work, we have obtained a fusion protein that consists of a combination of the* M. tuberculosis* CFP10 and ESAT6 antigens and human deltaferon. Our results suggest that the components of fusion protein expressed in carrot cells retain their antigenic properties. However, the expression level of the fusion protein in the storage roots of transgenic carrot plants in these studies is insufficient for a commercial product [43].

The experiments with laboratory animals have demonstrated that the fusion protein CFP10-ESAT6-dIFN is able to induce both humoral (Figure 3) and cell-mediated (Figure 4) immune responses when administered orally or by injection to warm-blooded animals (mice). We have previously demonstrated that individual rESAT6 is toxic to peripheral blood mononuclear cells [21] as well as transgenic plants at the stage of regeneration (causing various morphological abnormalities in regenerants). The immunization with rESAT6 or rCFP10 induced a statistically significant decrease in the SI by nonspecific inducers (ConA, PHA, Figure 5) as compared with the control, whereas statistically significant differences were not observed in the case of the fusion protein. Thus the* M. tuberculosis* antigens contained in the fusion protein have no cytotoxic effect on the peripheral blood mononuclear cells. Of note, the addition of the fusion protein or its component deltaferon stimulates proliferation of splenocytes in unimmunized mice (Figures 4 and 6).

A lower level of antibodies to orally administered rCFP10-ESAT6-dIFN compared with injection delivery is potentially explainable by two factors. First, it is possible that the cell wall of carrot cells is poorly destroyed in the gastrointestinal tract [44, 45] and the protein amount released by preliminary carrot mincing is insufficient for induction of a high immune response. Alternatively, the acids and digestive enzymes of the gastrointestinal tract may destroy the immunogenic protein. Interestingly, the transgenic carrot storage roots containing the fusion protein CFP10-ESAT6-dIFN do not differ from the efficiency of injected recombinant fusion protein in their ability to induce cell-mediated immunity. This is especially important because the Th1 type of immune response plays a leading role in resistance to* M. tuberculosis* infection [46, 47].

A Western blot assay for the fusion CFP10-ESAT6-dIFN protein detected several bands on the lanes loaded with the total protein of transgenic carrot storage roots and with purified recombinant fusion protein from* E. coli* (Figure 2(b)). The polyclonal serum for visualizing these proteins has been raised against the recombinant antigen rESAT6; correspondingly, all the molecules containing this polypeptide are detected by Western blot assay. During the extraction of proteins from plants, denaturing and reducing agents disrupt the trimeric and other oligomeric forms of fusion protein into the monomeric form. Under these conditions, fusion proteins are exposed to proteases and could result in cleavage [7]. And it is known that the proteins synthesized in a plant expression system can be posttranslationally modified [48]. Our experimental data are insufficient to determine the causes of the formation of low molecular weight bands. However we assume that in plant cells fusion protein remains in the mono- or multimeric form, since we did not observe the toxic effect of individual ESAT6 protein in animals immunized with fusion protein.

Summing up, the novel fusion protein rCFP10-ESAT6-dIFN is of clear interest for further studies. The next stages in our work are improvement of the genetic construct aimed at an increase in the expression level of the fusion recombinant protein in transgenic plant tissues, modification of its oral delivery that would provide for a better humoral immune response, and kinetic and a more detailed analysis of the immune response developed as a result of vaccination protocol in humanized animal model.

## 5. Main Conclusion

Fusion protein comprising the* M. tuberculosis* genes* cfp10* and* esat6* and* dIFN* gene, expressed in transgenic carrot, induces humoral and cell-mediated immune responses when administered orally or by injection.

## Abbreviations

ANOVA: Analysis of variance
SI: Stimulation index
LTT: Lymphocyte transformation test
EIA: Enzyme immunoanalysis.
